# The impact of perceived exertion on satisfaction with life among power line workers

**DOI:** 10.1192/j.eurpsy.2023.936

**Published:** 2023-07-19

**Authors:** I. Sellami, A. Feki, A. Abbes, M. A. Ghrab, M. L. Masmoudi, S. Baklouti, K. Jmal Hammami, M. Hajjaji

**Affiliations:** 1 Occupational medecine; 2 Rheumatology, Hedi Cahker Hospital, University of Sfax; 3 occupational medecine, Hedi Chaker Hospital, Sfax, Tunisia

## Abstract

**Introduction:**

The power line workers have a heavy physical workload. It is essential to know the impact of this perceived exertion on their satisfaction with life to improve their mental health.

**Objectives:**

We aimed to evaluate the associations between perceived exertion at work and satisfaction with life among power line workers.

**Methods:**

We conducted a study among a group of power line workers from January to June 2022 using a self-administered questionnaire. We evaluated socio-professional characteristics, physical exertion with the Borg CR-10, and the satisfaction with life scale (SWLS).

**Results:**

Seventy-four male line workers participated in the study. They were married in 67.6% of cases. The mean age was 39.3 ± 10.5 years. The average job tenure was 15.5 ± 11.2 years. The mean of perceived exertion was 6.1±1.9. High to very high exertion was found in 73% of participants. The mean score of satisfaction with life was 26.8 ±6.5. Five (12.6%) participants were dissatisfied to extremely dissatisfied. Fifty-six (75.9%) participants were satisfied to extremely satisfied. High perceived exertion was correlated with higher satisfaction with the lives of line workers (p = 0.03, r = 0.24).

**Conclusions:**

Power line workers with high perceived exertion were more satisfied with their lives. This can be explained by the positive impact of work on the lives of workers. The work environment is paramount to ensuring good mental health.

**Disclosure of Interest:**

None Declared

